# Supplementary material for: Effectiveness of Guided Breathing and Social Support for the Reduction of Pre-Exam Anxiety in University Students: A Factorial Study
Source: Healthcare (Basel). 2023 Feb 15;11(4):574. doi: 10.3390/healthcare11040574 (PMC9957036; doi:10.3390/healthcare11040574)
Supplement: Supplementary file 1 [file healthcare-11-00574-s001.zip › Suplementary file S1.pdf]

Escala GTAI-A

Marca con una X o rodea con un círculo la puntuación más acorde a tu sentimiento con respecto a la realización del presente examen.

Sexo: **Curso académico:**

Edad:

	**Casi nunca**	**En ocasiones**	**A menudo**	**Casi siempre**
1. Tengo seguridad en mi capacidad	**1**	**2**	**3**	**4**
2. Pienso en la importancia que tiene el examen para mi	**1**	**2**	**3**	**4**
3.Tengo una sensación rara en mi estomago	**1**	**2**	**3**	**4**
4.De repente se me cruzan pensamientos por la cabeza que me bloquean	**1**	**2**	**3**	**4**
5.Me preocupa saber si podré afrontarlo	**1**	**2**	**3**	**4**
6.Siento mi cuerpo tensionado	**1**	**2**	**3**	**4**
7.Tengo confianza en mi propio desempeño	**1**	**2**	**3**	**4**
8.Pienso en las consecuencias de fracasar	**1**	**2**	**3**	**4**
9.Me pregunto si mi rendimiento será lo suficientemente bueno	**1**	**2**	**3**	**4**
10.Pienso en cualquier cosa y me distraigo	**1**	**2**	**3**	**4**
11.Me siento incómodo/a	**1**	**2**	**3**	**4**
12. Sé que puedo confiar en mí mismo/a	**1**	**2**	**3**	**4**
13.Pienso en lo mucho que me importa obtener un buen resultado	**1**	**2**	**3**	**4**
14.Fácilmente pierdo el hilo de mis pensamientos	**1**	**2**	**3**	**4**
15.Siento que mi corazón me late fuerte	**1**	**2**	**3**	**4**
16.Me preocupo por el resultado de mi examen	**1**	**2**	**3**	**4**
17. Me siento ansioso/a	**1**	**2**	**3**	**4**
18.Otros problemas personales me interfieren y no me permiten recordar las cosas.	**1**	**2**	**3**	**4**
19.Me siento conforme conmigo mismo/a	**1**	**2**	**3**	**4**
20.Me preocupa cómo se verá mi calificación	**1**	**2**	**3**	**4**
21.Tiemblo de nerviosismo	**1**	**2**	**3**	**4**

22.Me preocupa que algo pueda salirme mal	**1**	**2**	**3**	**4**
23.Me llama la atención algo de poca importancia que interrumpe mi razonamiento	**1**	**2**	**3**	**4**
24.Tengo una sensación de angustia	**1**	**2**	**3**	**4**
25.Confío que lograre hacerlo todo	**1**	**2**	**3**	**4**
26.Pienso en lo que pasará si me va mal	**1**	**2**	**3**	**4**
27.Me siento nervioso/a	**1**	**2**	**3**	**4**
28.Estoy convencido de que haré bien el examen	**1**	**2**	**3**	**4**

GRACIAS POR TU PARTICIPACIÓN

Escala GTAI-A

Mark with an X or circle the score that most closely matches your feelings about taking this test.

Sex: **Academic Course:**

Age:

	Casi nunca	En ocasiones	A menudo	Casi siempre
1. I am confident in my ability	1	2	3	4
2. I think about how important the exam is to me.	1	2	3	4
3.I have a weird feeling in my stomach.	1	2	3	4
4.Suddenly thoughts cross my mind and block me.	1	2	3	4
5.I am worried about whether I will be able to cope with it.	1	2	3	4
6.I feel my body tense	1	2	3	4
7. I have confidence on my own performance	1	2	3	4
8.I think about the consequences of failure	1	2	3	4
9.I wonder if my performance will be good enough.	1	2	3	4
10.I think about anything and I get distracted.	1	2	3	4
11.I feel uncomfortable	1	2	3	4
12. I know I can trust myself	1	2	3	4
13.I think about how much I care about getting a good result.	1	2	3	4
14.I easily lose my train of thought.	1	2	3	4
15.I feel my heart pounding	1	2	3	4
16.I worry about my exam result.	1	2	3	4
17. I feel anxious	1	2	3	4
18.Other personal problems interfere with me and prevent me from remembering things.	1	2	3	4
19.I feel happy with myself	1	2	3	4
20.I worry about how my grade will look	1	2	3	4
21.I shake with nervousness	1	2	3	4
22.I worry that something might go wrong	1	2	3	4
23.My attention is drawn to something of little importance that interrupts my reasoning	1	2	3	4
24.I have a feeling of anguish	1	2	3	4
25.I am confident that I will get everything done	1	2	3	4

26.I think about what will happen if I do not do well.	**1**	**2**	**3**	**4**
27.I feel nervous	**1**	**2**	**3**	**4**
28.I am convinced that I will do well in the exam.	**1**	**2**	**3**	**4**
